# Illusory face detection in pure noise images: The role of interindividual variability in fMRI activation patterns

**DOI:** 10.1371/journal.pone.0209310

**Published:** 2019-01-14

**Authors:** Kristin M. Zimmermann, Ann-Sophie Stratil, Ina Thome, Jens Sommer, Andreas Jansen

**Affiliations:** 1 Department of Psychiatry, University of Marburg, Marburg, Germany; 2 Center for Mind, Brain and Behavior (CMBB), University of Marburg, Marburg, Germany; 3 Core-Unit Brainimaging, Faculty of Medicine, University of Marburg, Marburg, Germany; The University of Melbourne, AUSTRALIA

## Abstract

Illusory face detection tasks can be used to study the neural correlates of top-down influences on face perception. In a typical functional magnetic resonance imaging (fMRI) study design, subjects are presented with pure noise images, but are told that half of the stimuli contain a face. The illusory face perception network is assessed by comparing blood oxygenation level dependent (BOLD) responses to images in which a face has been detected against BOLD activity related to images in which no face has been detected. In the present study, we highlight the existence of strong interindividual differences of BOLD activation patterns associated with illusory face perception. In the core system of face perception, 4 of 9 subjects had highly significant (*p*<0.05, corrected for multiple comparisons) activity in the bilateral occipital face area (OFA) and fusiform face area (FFA). In contrast, 5 of 9 subjects did not show any activity in these regions, even at statistical thresholds as liberal as *p* = 0.05, uncorrected. At the group level, this variability is reflected by non-significant activity in all regions of the core system. We argue that these differences might be related to individual differences in task execution: only some participants really detected faces in the noise images, while the other subjects simply responded in the desired way. This has several implications for future studies on illusory face detection. First, future studies should not only analyze results at the group level, but also for single subjects. Second, subjects should be explicitly queried after the fMRI experiment about whether they really detected faces or not. Third, if possible, not only the overt response of the subject, but also additional parameters that might indicate the perception of a noise stimulus as face should be collected (e.g., behavioral classification images).

## Introduction

Face-processing is mediated by a distributed, typically right-lateralized neural network. This network is often divided into a *core system* and an *extended system* [1,2].he core system is engaged in processing basic information about faces. It consists of brain regions in the occipito-temporal cortex: the fusiform face area (FFA), located in the fusiform gyrus; the occipital face area (OFA), located in the inferior occipital gyrus; and an area in the posterior superior temporal sulcus (pSTS). Each region plays a different role in face processing. The OFA is thought to be responsible for the early processing of physical features of face stimuli and sends its output to both the FFA and pSTS. The FFA is associated with the representation of invariant aspects of the face (e.g. face identity), while the pSTS processes changeable aspects of faces (e.g., facial expression, direction of eye-gaze, lip movements). Beyond the core system, there are a number of additional (not face-preferential) regions that contribute to face processing, e.g., the inferior frontal gyrus (IFG), the amygdala, the insula, and the orbitofrontal cortex (OFC) [3]. This extended system of face processing comes into play when additional information is extracted from faces, e.g., emotions, biographic information, attractiveness [2,4]. In particular, the amygdala and the insula are associated with the processing of facial expressions [5], the IFG with the analysis of face-related semantic aspects [6,7], the OFC and the nucleus accumbens with the processing of facial beauty or sexual relevance [8,9] (for a critical evaluation of the model, see also e.g., [10,11]).

Interactions between brain regions of the face perception network can be broadly divided into bottom-up processing and top-down modulations. On the one hand, sensory input from the primary visual cortex enters the core system in a bottom-up and feed-forward fashion and is subsequently passed to more anterior regions of the extended system. On the other hand, higher order brain regions exert top-down modulations on regions located in the core system, for instance, in order to use existing knowledge about faces for the interpretation of ambiguous sensory input [12]. Importantly, interactions between bottom-up and top-down processes are ubiquitously present in the visual system and already occur at very early levels, as e.g., in the lateral geniculate nucleus (LGN; [13]). However, over the past years, sensory processing of faces has been studied extensively, whereas the neural mechanisms of top-down face processing are still poorly understood.

In neuroimaging studies, different paradigms have been developed to investigate top-down modulations, such as instructing subjects to imagine faces [14,15], to interpret ambiguous face stimuli [16], to process noisy faces [17], or to identify illusory faces in pure noise images [18–20]. Illusory face detection paradigms have the advantage that top-down influences on face perception are particularly pronounced as stimuli do not include physical faces (but random noise). However, noise stimuli including face-like structures (i.e., coincidental dark parts in a physiologically plausible arrangement resembling eyes, nose and mouth) seem to increase the likelihood to be classified as faces (e.g., [18,21]). Hence, illusory face perception most likely relies on a strong top-down modulation of sensory, bottom-up input. One way to study illusory face detection is to compare pure noise images (i.e., pictures without faces) in which a face has supposedly been detected against those pure noise images in which no face has been detected. In a series of four consecutive studies, Zhang, Liu, Li and colleagues investigated the neural correlates of illusory face perception (“face pareidolia”, [18–20]). They showed that face pareidolia was associated with BOLD activity in the bilateral OFA and FFA. Further, they suggested that the prefrontal cortex, in particular the OFC, might be the source of top-down modulations in illusory face perception.

In the present study, we aimed to use a similar experimental paradigm to investigate top-down modulated interactions of brain regions during illusory face perception. We originally planned to apply a bilateral neural network model to the neuroimaging data. Unlike previous studies, we aimed to disentangle differential contributions of left- and right-hemispheric brain regions of the core system of face perception (for a similar approach using bilateral neural models to analyze neuroimaging data on face processing, see [1,22,23]). It turned out, however, that we were not able to replicate the main findings from the previous studies [18–20], i.e., at the group level we did not find any BOLD activity in the core system of face perception when comparing events in which a face was detected against events in which no face was detected.

To understand this discrepancy, we also evaluated the data of individual subjects. It turned out that individual BOLD activity followed an almost dichotomous distribution. About half of the subjects showed significant activity in all regions of the core system (bilateral OFA, bilateral FFA), while the other half did not show any noteworthy activity at all, not even at liberal statistical thresholds (*p* = 0.05, uncorrected). These interindividual differences explained why we were not able to replicate previous findings at the group level (of note, this prevented us from finishing the originally planned analyses that would have required sufficiently strong activation at the single subject level in predefined regions-of-interest in more than half of the subjects).

We wrote the present article to highlight the consequences of interindividual differences in task execution during illusory face perception for the interpretation of BOLD activity. We believe that it is important to analyze brain imaging data for illusory face detection paradigms not only at the group level, but also at the individual subject level, as long as there is no additional marker (apart from the overt response) that indicates whether a noise stimulus was perceived as a face or not. This issue has, to our knowledge, not been discussed in previous publications on that topic.

As outlined above, the present article was motivated by the fact that we were only partly successful in replicating results from previous studies of another research group using a similar study design [18–20]. Interestingly, a more detailed analysis of these previous results also showed striking differences between studies, in particular with regard to brain activation of the core system of face perception. To give one example: Zhang et al. [19] explicitly reported that greater activation was revealed for face versus non-face responses in the fusiform face area, but not in the occipital face area. In contrast, both Li et al. [20] and Liu et al. [18] reported brain activation differences also in the OFA.

## Materials and methods

### Subjects

Nine subjects (3 male, age range 20–26 years) participated in the experiment. All subjects were healthy and had no history of neurological or psychiatric diseases, brain pathology or abnormal brain morphology on T1-weighted MR images. They gave informed written consent before the study. The study protocol was approved by the local ethics committee of the Medical faculty of the University of Marburg. All participants were right-handed according to the Edinburgh Inventory of Handedness [24].

### Experimental procedure

The experiment was divided into two parts. First, subjects performed an illusory face detection task in which they had to detect faces in pure noise images. Second, subjects performed a classical face localizer task to identify brain regions that were activated during real face perception. All stimuli were presented using the Presentation 11.0 software package (Neurobehavioral Systems, Albany, CA, USA, www.neurobs.com).

### Illusory face detection task

The illusory face detection paradigm was based on a task that has been used in a number of previous imaging studies [18–20]. It consisted of two stages: a training period and a test period. Both periods were performed inside the MR scanner. However, we were only interested in the analysis of the data of the test period. The training period was mainly included in the experiment to increase the likelihood that subjects detected faces in noise images by gradually enhancing the noise level. Five types of stimuli were used: face images overlaid with 10% noise (easy-to-detect faces), face images overlaid with 50% noise (difficult-to-detect faces), faces overlaid with 75% noise (more-difficult-to detect faces), pure noise images, and checkerboard images (serving as low-level baseline in the fMRI analysis; for a depiction of stimuli, see supplementary material, S1 Fig [19]).

The training period was divided into eight blocks that were separated by rest periods with a duration of 20 s. In each block, subjects were presented 28 trials. Each trial started with a fixation cross of 200 ms duration after which the stimulus was presented for 600 ms, followed by a black screen for 1200 ms. In the first six blocks, the trials consisted of ten face images overlaid with noise (block 1 and 2: 10% noise; block 3 and 4: 50% noise; block 5 and 6: 75% noise), ten pure noise images, and eight checkerboard images. In block 7 and 8, the trials consisted of 20 pure noise images and eight checkerboard images. The order of trials (face images, pure noise images, checkerboard images) was pseudo-randomized within each block. Subjects were instructed that 50% of the presented images contained a face, and 50% did not. They were told that the task would become more difficult over time and were instructed to press a button on a MR-compatible response-box with their right index finger whenever they saw a face.

The test period was divided into four blocks that were separated by rest periods with a duration of 20 s. In each block, subjects were shown 120 pure noise images and 40 checkerboard images in a pseudo-randomized order. The order of events in one trial was the same as during the training period. Again, subjects were told that 50% of the presented images contained a face, and 50% did not. They were instructed to press a button on a MR-compatible response-box with their right index finger whenever they saw a face.

### Face localizer task

After the illusory face detection task, a face localizer task was used to identify brain regions that were activated during face perception. The localizer task consisted of nine blocks lasting 16 s each. Blocks were separated by 8 s rest periods in which a fixation cross was presented. Each block consisted of 16 trials in which a stimulus of a specific stimulus category was presented for 600 ms, followed by a fixation cross for 400 ms. Three stimulus categories were used: faces, objects, and scrambled images (i.e., Fourier transforms of the face and object stimuli). Three blocks of each category were shown in pseudo-randomized order. In each block, two randomly chosen stimuli contained a red dot. Subjects were instructed to press a button on a MR-compatible response-box with their right index finger when they saw the red dot. These “catch” trials were used to ensure continuous attention to the task.

### MRI data acquisition

All MRI data were acquired on a 3-Tesla Tim Trio MR scanner (Siemens Medical Systems) with a 12 channel head coil at the Department of Psychiatry, University of Marburg. Functional images were collected with a T2*-weighted echo planar imaging (EPI) sequence sensitive to the BOLD contrast (64x64 matrix, FOV 200 mm, in plane resolution 3.13 mm, 36 slices, slice thickness 3 mm, TR = 2.2 s, TE = 30 ms, flip angle 90°). Slices covered the whole brain and were positioned transaxially parallel to the anterior-posterior commissural line (AC-PC). The initial 3 images were excluded from further analysis in order to remove the influence of T1 stabilization effects. For each subject, we additionally acquired a high-resolution anatomical image using a T1-weighted magnetization-prepared rapid gradient-echo (3d MP-RAGE) sequence in sagittal plane (176 slices, TR = 1900 ms, TE = 2.26 ms, matrix size 256×256 voxels, voxel size 1×1×1 mm, flip angle 9°).

### Data analyses

SPM8 (http://www.fil.ion.ucl.ac.uk/spm) standard routines and templates were used for the analysis of fMRI data. The functional images were realigned, normalized (resulting voxel size 2x2x2 mm^3^), smoothed (8 mm isotropic Gaussian filter) and high-pass filtered (cut off period 128 s). Statistical analysis was performed in a two-level, mixed-effects procedure.

### Illusory face detection task (test period)

Based on the behavioral data, the noise images were classified as “face”, “no face”, or “no response”. At the individual subject level, BOLD responses for each condition (face, no face, no response, checkerboard) were modeled with the canonical hemodynamic response function. The six realignment parameters were included as nuisance regressors. Using the parameter estimate images, t-contrast images were calculated for the contrast “face > no face”. At the group level, the contrast images for the contrast “face > no face” were entered into a one-sample t-test.

### Face localizer task

At the individual subject level, the BOLD responses for the three conditions (faces, objects, scrambled images) were modeled by a boxcar function convolved with the canonical hemodynamic response function. The six realignment parameters were included as nuisance regressors. Using the parameter estimate images, a t-contrast image was calculated for the contrast “2*faces > (objects + scrambled images)”. At the group level, the contrast images were entered into a one-sample t-test. The anatomical localization of activated brain regions was assessed both by the SPM anatomy toolbox [25] and the WFU-Pickatlas [26].

### Analysis strategy

First, we analyzed the data from the face localizer task. Using the contrast “2*faces > (objects + scrambled images)”, we identified for each subject the localization of the right FFA, left FFA, right OFA, and left OFA. Using the WFU-Picklatlas, we then created individual spherical masks (radius 6 mm) at the corresponding local maxima of the t-map (MNI space). These individually-tailored masks were subsequently used for region-of-interest (ROI) analyses of the illusory face detection task. Second, we analyzed the data from the illusory face detection task. We used the contrast “face > no face” to investigate whether the detection of faces in pure noise images activates brain regions in the core system of face perception (OFA, FFA). We performed both whole brain analyses and ROI analyses. For the ROI analysis, we used the individually-tailored masks of bilateral OFA and FFA, respectively, as described above.

## Results

### Face localizer task

**Fig 1:**
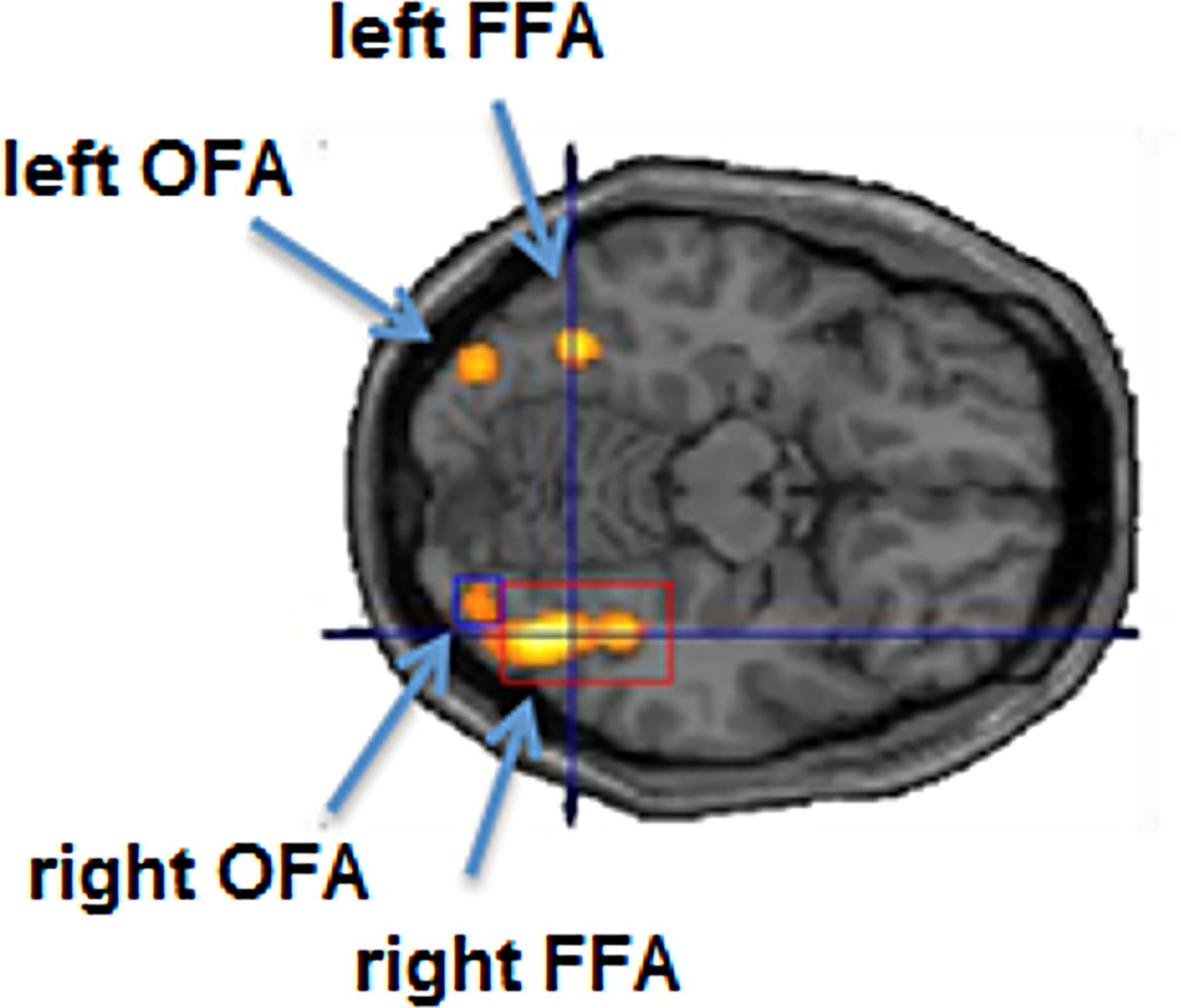
Activation pattern of a representative subject (S1) for the face localizer task (contrast: “2*faces > (objects + scrambled images)”; *p* = 0.05, corrected (FWE) for multiple comparisons, transversal section at z = -14 mm).

At the group level, we found right-lateralized BOLD activity in the right and left FFA and the right and left OFA at *p* = 0.001, uncorrected for multiple comparisons (Table 1). At the individual subject level, all 9 subjects showed activation in the right FFA and OFA at *p* = 0.001, uncorrected for multiple comparisons. 7 of 9 subjects showed activity in the left FFA, 8 of 9 subjects activity in the left OFA (see Fig 1 for the activation pattern of a representative subject). When the statistical threshold was further relaxed to *p* = 0.1, uncorrected, we found activity in the left FFA for subject S3 (see Table 1). No activation peak could be detected for the left OFA for subject S3 and for the left FFA for subject S4. The peak coordinates of all activations are listed in Table 1. They were consistent with the loci of FFA and OFA activity reported in previous studies of face processing (e.g., [1,27]).

**Table 1:** Activation maxima (reported in MNI coordinates) in regions of the core system of face perception for the face localizer task (contrast “2*faces > (objects + scrambled images)”, *p* = 0.001, uncorrected for multiple comparisons; * *p* = 0.1) for the group analysis (GA) and the individual subject analysis (S1-S9).

	Right FFA			Left FFA			Right OFA			Left OFA		
	**x**	**y**	**z**	**x**	**y**	**z**	**x**	**y**	**z**	**x**	**y**	**z**
**GA**	40	-40	-20	-40	-46	-20	32	-88	-12	-40	-84	-10
**S1**	38	-48	-20	-36	-60	-14	44	-86	-6	-32	-86	-16
**S2**	36	-64	-14	-38	-54	-24	32	-90	-12	-34	-78	-12
**S3**	42	-50	-26	-40	-64	-18*	40	-80	-10	-	-	-
**S4**	38	-60	-20	-	-	-	40	-82	-8	-42 -36	-80 -86	-6 -8
**S5**	44 40	-52 -38	-20 -16	-48	-58	-18	46	-76	-4	-42	-86	-10
**S6**	44	-54	-24	-44	-60	-24	48	-78	-4	-40	-86	-2
**S7**	44	-48	-22	-36	-80	-14	38	-84	-8	-46 -48	-74 -60	-6 -18
**S8**	38	-42	-26	-36	-62	-18	24	-96	-6	-40	-78	-14
**S9**	38 42	-32 -60	-18 -22	-38	-58	-18	38	-84	-10	-36	-84	-8

### Illusory face detection task

**Fig 2:**
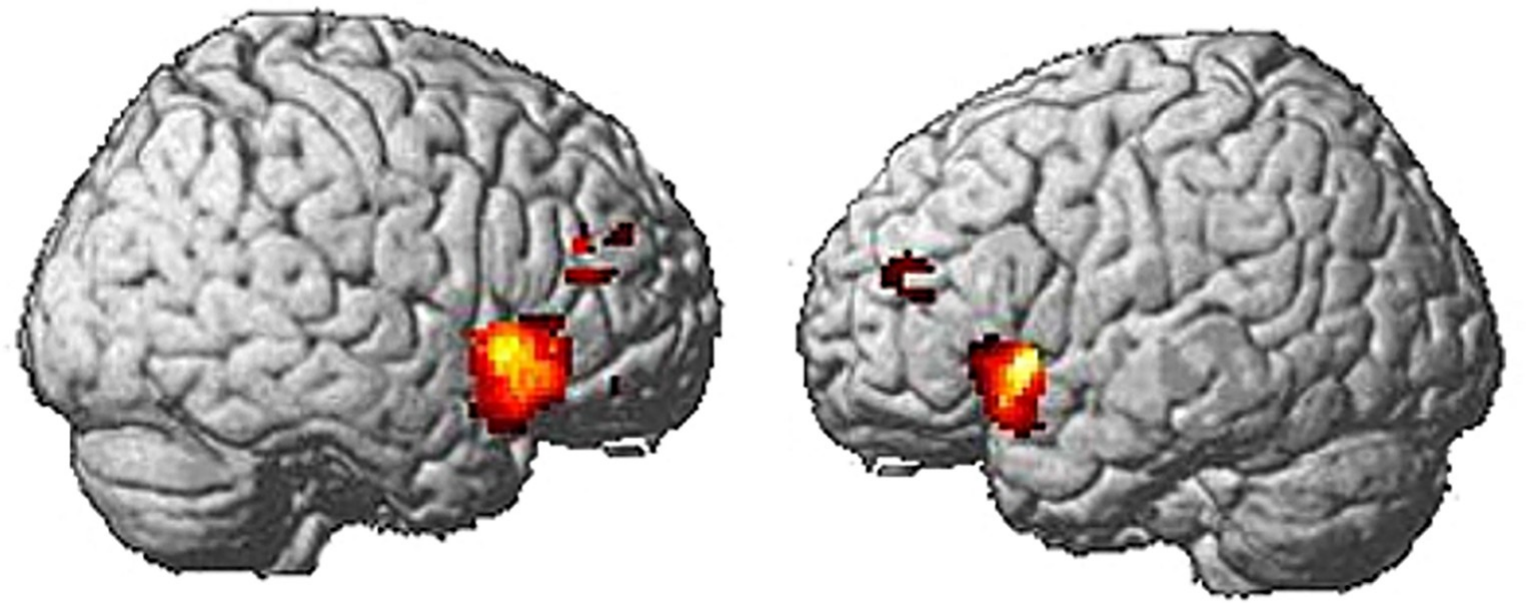
Group activation pattern for the illusory face detection task (contrast “face > no face”, *p* = 0.001 uncorrected. BOLD activation was found in prefrontal and parietal regions, but not in the core system of face perception (e.g., OFA and FFA).

On average, subjects indicated that they saw a face in 23.5% of the noise images (standard deviation 11.4%, range 9.2% - 44.0%). Based on these classifications, we analyzed whether we were able to detect BOLD activity for the contrast “face > no face” in the core system of face perception (OFA, FFA). At the group level, we found significant BOLD activity in a left-lateralized fronto-parietal network, but not in occipito-temporal regions (*p* = 0.001, uncorrected at the voxel level, *p* = 0.1, corrected (FDR) for multiple comparisons at the cluster level) (Fig 2). Only when we relaxed the statistical threshold to *p* = 0.05, uncorrected, we also detected activity in the core-system of face perception (OFA, FFA).

**Fig 3:**
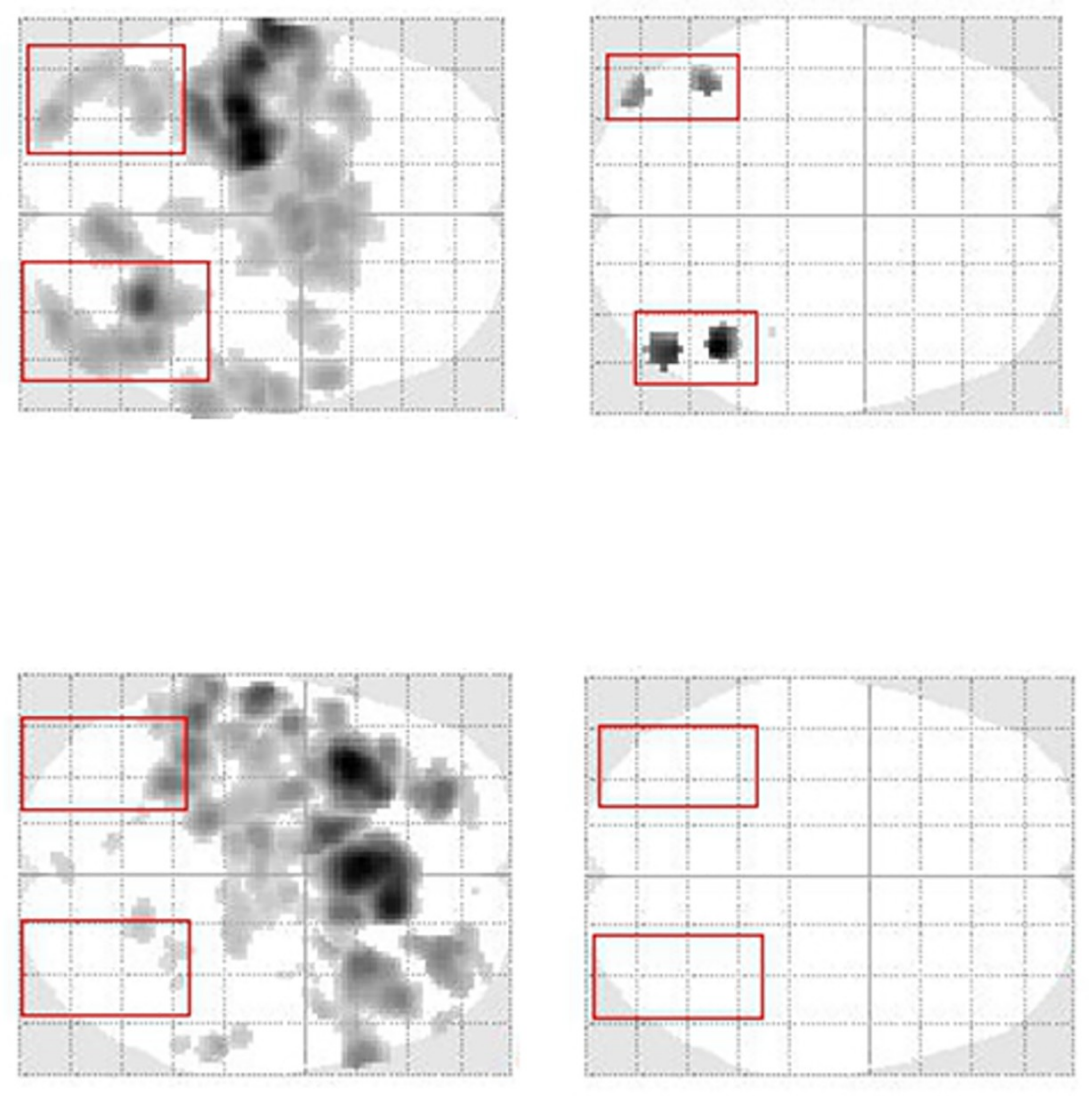
Representative BOLD activation pattern during the illusory face detection task for two subjects (contrast: “face > no face”). **Subject S5 (top) shows significant BOLD activity in the core-system of face perception, as assessed either by a whole brain analysis (left; *p* = 0.001, uncorrected) or a ROI analysis (right; *p* = 0.05, corrected (FWE) for multiple comparisons). In contrast, subject S2 (bottom) does not show BOLD activity in the ROIs of the core-system of face perception, not even at the liberal threshold of *p* = 0.05, uncorrected.** Left: whole brain analysis, right: ROI analysis.

**Fig 4:**
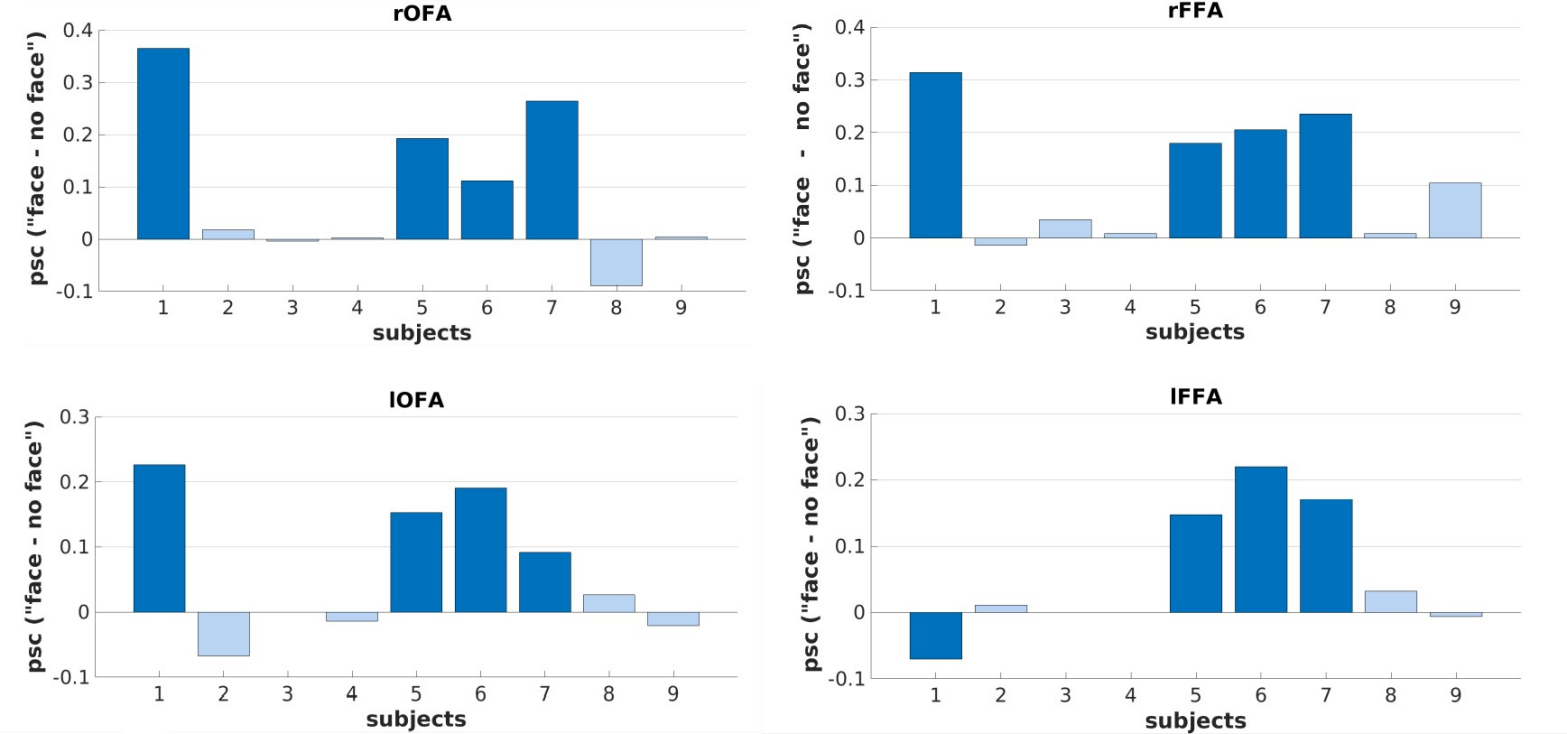
Difference of mean percent signal change (psc) over the respective ROIs of the core system of face perception (right OFA, right FFA, left OFA, left FFA) between the conditions “face” and “no face” for individual subjects. Subjects with significant BOLD activity in the ROI analysis (1, 5, 6, 7) are colored in dark blue, subjects without significant BOLD activity in the ROI analysis (2, 3, 4, 8, 9) are colored in light blue. Psc was calculated using MarsBaR (marsbar.sourceforge.net/).

At the individual subject level, we detected large interindividual differences with regard to BOLD activation in the core system of face perception (i.e., OFA, FFA). 4 of 9 subjects (S1, S5, S6, S7) showed strong activity in all four ROI masks, both in a whole brain analysis (*p* = 0.001, uncorrected at the voxel level, cluster threshold *k* = 10) as well as in a ROI analysis (*p* = 0.05, corrected (FWE) for multiple comparisons). In contrast, 5 of 9 subjects (S2, S3, S4, S8, S9) did not show activity in any of the FFA- and OFA-ROI masks, even when the threshold was relaxed to *p* = 0.05, uncorrected. Importantly, we found no evidence for an association of this finding with the individual percentage of events classified as “face” (see S1 Table). BOLD activation patterns for two representative subjects are depicted in Fig 3. In the supplementary material, we additionally show the BOLD activation patterns for all subjects (S2 Fig). Fig 4 illustrates the mean percent signal changes for each ROI and each individual separately.

Although interindividual variability is a common finding in fMRI, however, the present results are exceptional insofar as we do not find the typically expected approximately Gaussian distributed variations in the effect size of brain activity, but rather an almost dichotomous distribution. Hierarchical cluster analyses on the subject’s mean percent signal changes of, on the one hand all ROIs (excluding subject S3 and S4 because of missing values) and, on the other hand ROIs of the right hemisphere (including all subjects) further support this assumption by revealing trends towards a two-group structure (see S3 Fig). After we had analyzed the data, we hypothesized that this finding might be caused by the fact that only some participants detected faces in the noise images and the other subjects simply responded in the desired way. Since several months had passed since the experiment, it was not possible to post-hoc interview all subjects. Nevertheless, we were able to query three subjects that did not show any activity in the core system. Two of them explicitly confirmed our hypothesis that they indeed did not see any faces in the noise images, but pressed the ‘face detected’ button because they thought they were expected to see a face.

## Discussion

Illusory face detection tasks can be used to study the neural correlates of top-down influences on face perception by comparing BOLD activity associated with the perception of pure noise images in which a face has supposedly been detected against BOLD activity related to pure noise images in which no face has been detected. In the present study, we highlighted the existence of strong interindividual differences of activation patterns associated with illusory face perception. In the core system of face perception, 4 of 9 subjects had significant activity (*p*<0.05, corrected (FWE) for multiple comparisons) in the bilateral OFA and FFA. In contrast, 5 of 9 subjects did not show any activity in these regions, even at statistical thresholds as liberal as *p* = 0.05, uncorrected. At the group level, this variability is reflected by non-significant activity in all regions of the core system.

At first glance, one might argue that interindividual variability is a common finding in fMRI. While this is certainly true, the present results do not show the expected Gaussian distribution in the effect size of brain activity, but a rather dichotomous distribution. This clear-cut dissociation might arise from interindividual differences in task execution. A possible explanation for these differences, as confirmed by the feedback given by some subjects, is that only some participants really felt that they detected faces in the noise images. The other subjects just gave the desired answer (“I did not see any faces in those images, but I pressed the ‘face detected’ button anyway because you expected me to see faces”, is an exemplary feedback one of the subjects gave after the experiment.).

In general, the drawback of illusory face detection paradigms is that one typically has to rely solely on the overt response of a subject when classifying a noise stimulus as “face” or “no face” event. One does therefore not know whether a participant felt like having seen a face or whether she simply gave a desired answer. This has several implications for future studies on illusory face detection. First, future studies should take interindividual variability more into account when presenting results. In particular, BOLD activation results should not only be analyzed at the group level, but also for single subjects. This information might be helpful to understand, for instance, why previous results are not fully replicated by follow-up studies. Second, subjects should be explicitly queried after the fMRI experiment whether they really detected faces or not. Third, if possible, not only the overt response of a subject, but also additional parameters that might indicate the perception of a noise stimulus as face should be collected. It is possible, for example, to conduct a reverse correlation analysis and calculate behavioral classification images. Here, all “face detected” stimuli and “no face detected” stimuli, respectively, are summed up and the difference between both sums of images is calculated. In order to increase signal-to-noise ratio and to uncover prominent structures, classification images can further be smoothed. Often, a successful induction of face pareidolia is associated with classification images showing facial features and thus representing subject’s internal representation of a facial structure (for an example, see [12]). It must be noted, however, that a much greater number of stimuli is needed compared to the amount of stimuli or trials typically used in fMRI experiments (e.g., Smith et al. [12] used 10,500 random noise images compared to 480 stimuli shown in the present study). In the present study, we therefore were not able to see any facial features in the resulting classification images.

In conclusion, we showed that illusory face detection paradigms can be associated with high interindividual variability at the BOLD activation level. Future studies on illusory face detection should take interindividual variability more into account when presenting neuroimaging results.

## Supporting information

S1 Fig Examples of the stimulus material used in the training and test periods of the experiment. The first three images (upper left, upper right and lower left) show a face overlaid with different degrees of noise. The last image (lower right) shows pure noise. During the test period of the study, only pure noise images (without overlaid faces) and checker board images were presented.
(TIF)

S2 Fig BOLD activation patterns during the illusory face detection task (contrast: “face”—“no face”; left: axial glass brain, whole brain analysis, *p* = 0.001, uncorrected; right: axial glass brain, *p* = 0.05, corrected (FWE) for multiple comparisons) of all nine subjects (S1 –S9). All subjects with significant BOLD activity in the ROI analysis (S1, S5, S6, S7) are shown in the left, subjects with no significant BOLD activity in the ROI analysis (S2, S3, S4, S8, S9) in the right column. Grey bars illustrate peak voxel contrast estimates (c.e.) and 90% confidence intervals of the respective ROIs of the core system of face perception (right OFA: bottom left; right FFA: bottom right; left OFA: top left; left FFA: top right). The left bar shows the c.e. of the “no face” condition and right bar shows the c.e. of the “face” condition.
(PDF)

S3 Fig Dendrograms obtained from the hierarchical cluster analyses using Wards method. To identify homogeneous subgroups of the sample by variation patterns of mean percent signal changes (psc) (i.e., differences of psc during “face” and “no face” trials, see Fig 4), we conducted hierarchical cluster analyses utilizing Wards method of minimum variance with a squared Euclidean distance measure in SPSS 24, (A) Cluster analysis using all ROIs (right OFA, right FFA, left OFA, left FFA), without subjects S3 and S4 because of missing cases. (B) Cluster analysis using ROIs of the right hemisphere (right OFA, right FFA), including all subjects. Based on the two dendrograms, we suggest a trend towards two clusters: Subjects with no significant BOLD activity in the ROI analysis were grouped (light blue) whereas in both analyses subjects with significant BOLD activity in the ROI analysis form a different cluster (dark blue). Mean psc over the respective ROI was assessed using MarsBaR (marsbar.sourceforge.net/).
(TIF)

S1 Table Results of correlation analyses between the individual percentage of events classified as “face” and the difference of psc between the conditions “face” and “no face” for individual subjects. (TIF)
