# Supplementary figures and images for: Illusory face detection in pure noise images: The role of interindividual variability in fMRI activation patterns
Source: PLoS One. 2019 Jan 14;14(1):e0209310. doi: 10.1371/journal.pone.0209310 (PMC6331101; doi:10.1371/journal.pone.0209310)

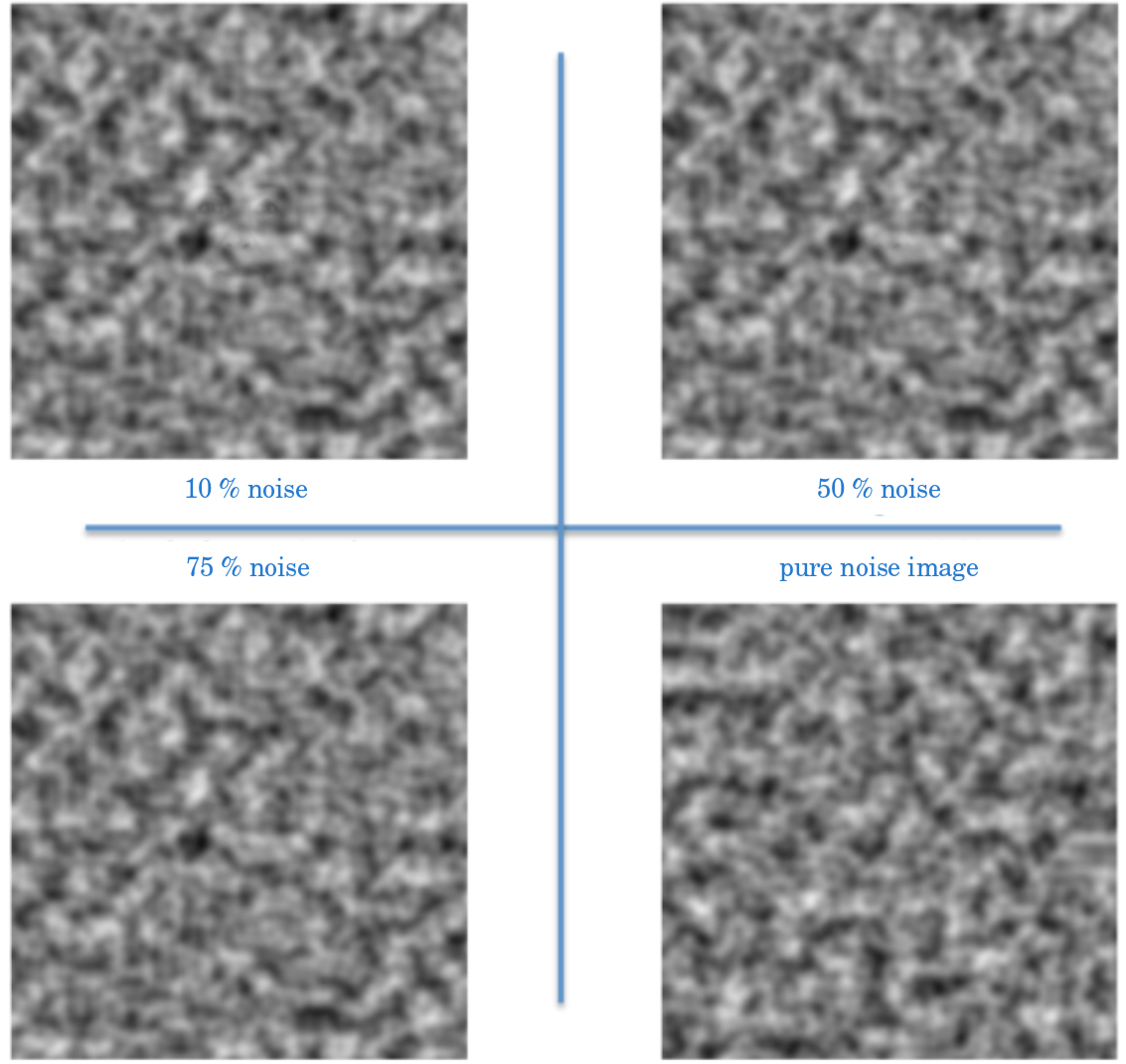

Supplement: S1 Fig — The first three images (upper left, upper right and lower left) show a face overlaid with different degrees of noise. The last image (lower right) shows pure noise. During the test period of the study, only pure noise images (without overlaid faces) and checker board images were presented. (TIF) [file pone.0209310.s001.tif]

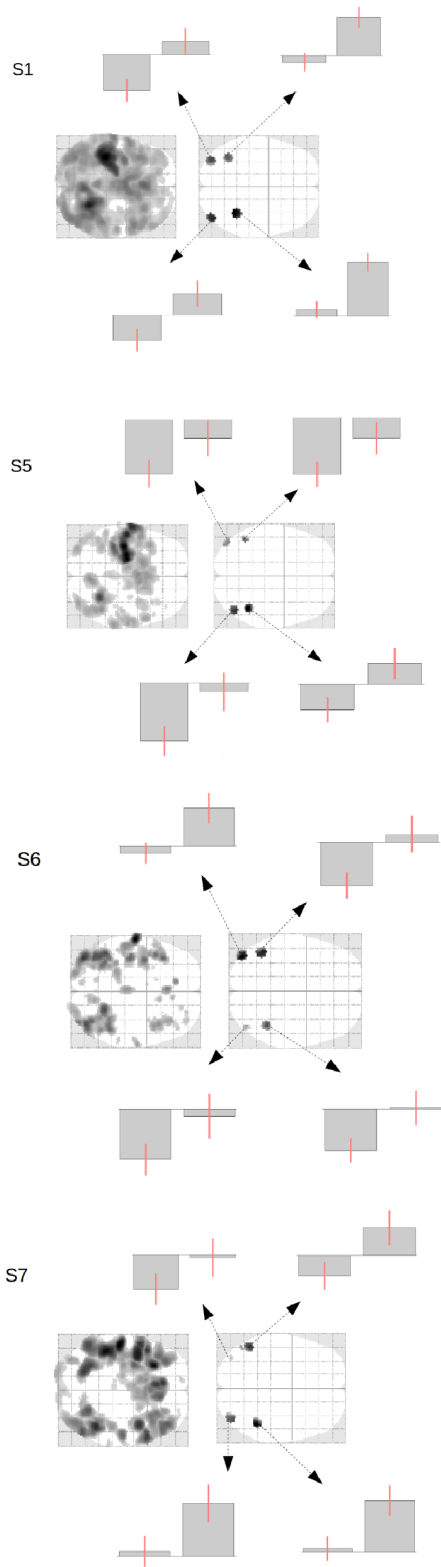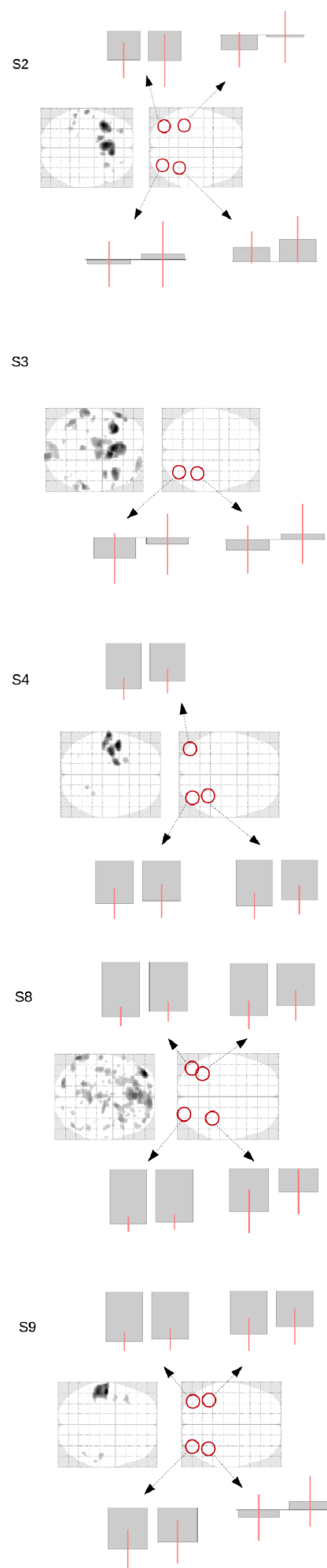

Supplement: S2 Fig — All subjects with significant BOLD activity in the ROI analysis (S1, S5, S6, S7) are shown in the left, subjects with no significant BOLD activity in the ROI analysis (S2, S3, S4, S8, S9) in the right column. Grey bars illustrate peak voxel contrast estimates (c.e.) and 90% confidence intervals of the respective ROIs of the core system of face perception (right OFA: bottom left; right FFA: bottom right; left OFA: top left; left FFA: top right). The left bar shows the c.e. of the “no face” condition and right bar shows the c.e. of the “face” condition. (PDF) [file pone.0209310.s002.pdf]

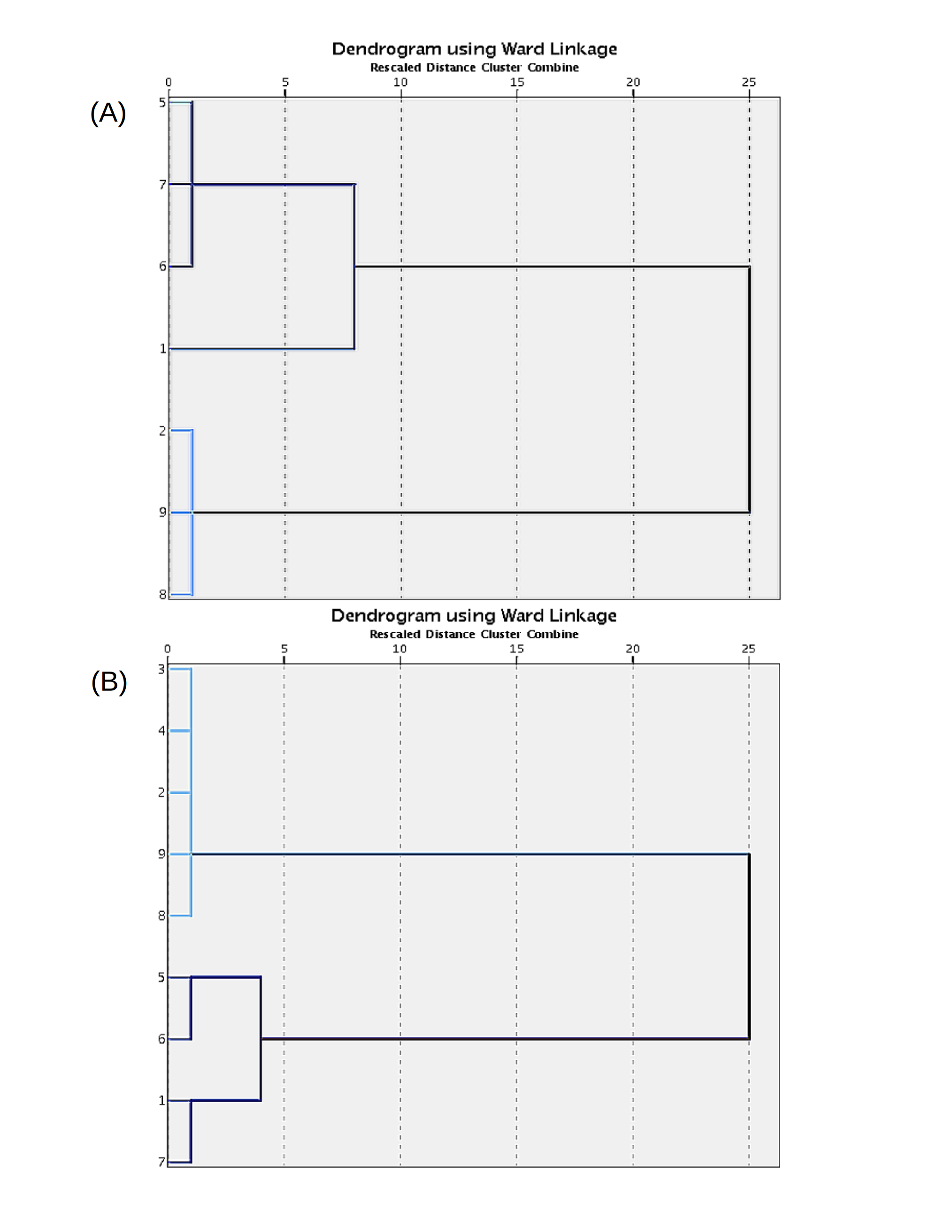

Supplement: S3 Fig — To identify homogeneous subgroups of the sample by variation patterns of mean percent signal changes (psc) (i.e., differences of psc during “face” and “no face” trials, see Fig 4), we conducted hierarchical cluster analyses utilizing Wards method of minimum variance with a squared Euclidean distance measure in SPSS 24, (A) Cluster analysis using all ROIs (right OFA, right FFA, left OFA, left FFA), without subjects S3 and S4 because of missing cases. (B) Cluster analysis using ROIs of the right hemisphere (right OFA, right FFA), including all subjects. Based on the two dendrograms, we suggest a trend towards two clusters: Subjects with no significant BOLD activity in the ROI analysis were grouped (light blue) whereas in both analyses subjects with significant BOLD activity in the ROI analysis form a different cluster (dark blue). Mean psc over the respective ROI was assessed using MarsBaR (marsbar.sourceforge.net/). (TIF) [file pone.0209310.s003.tif]

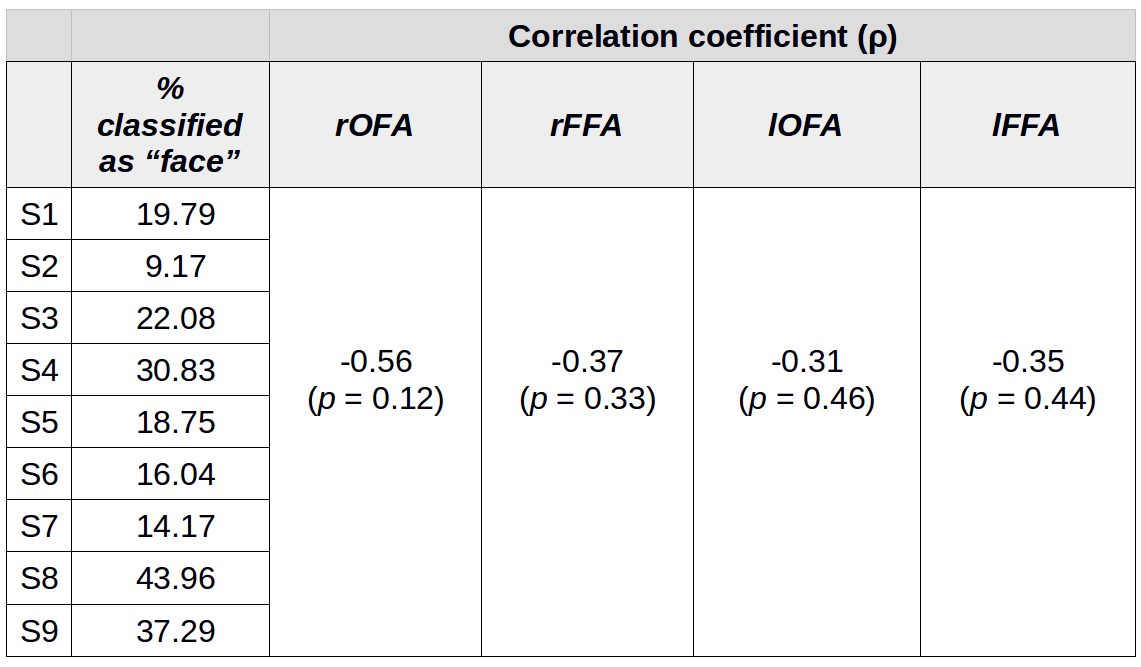

Supplement: S1 Table — (TIF) [file pone.0209310.s004.tif]
